# Differential role of ICAM ligands in determination of human memory T cell differentiation

**DOI:** 10.1186/1471-2172-8-2

**Published:** 2007-01-18

**Authors:** Omar D Perez, Dennis Mitchell, Garry P Nolan

**Affiliations:** 1 The Baxter Laboratory for Genetic Pharmacology, Stanford University School of Medicine, Stanford, CA 94305, USA; 2 Department of Microbiology and Immunology, Stanford University School of Medicine, Stanford, CA 94305, USA

## Abstract

**Background:**

Leukocyte Function Antigen-1 (LFA-1) is a primary adhesion molecule that plays important roles in T cell activation, leukocyte recirculation, and trans-endothelial migration. By applying a multivariate intracellular phospho-proteomic analysis, we demonstrate that LFA-1 differentially activates signaling molecules.

**Results:**

Signal intensity was dependent on both ICAM ligand and LFA-1 concentration. In the presence of CD3 and CD28 stimulation, ICAM-2 and ICAM-3 decreased TGFβ1 production more than ICAM-1. In long-term differentiation experiments, stimulation with ICAM-3, CD3, and CD28 generated IFNγ producing CD4+CD45RO+CD62L-CD11a^Bright^CD27- cells that had increased expression of intracellular BCL2, displayed distinct chemokine receptor profiles, and exhibited distinct migratory characteristics. Only CD3/CD28 with ICAM-3 generated CD4+CD45RO+CD62L-CD11a^Bright^CD27- cells that were functionally responsive to chemotaxis and exhibited higher frequencies of cells that signaled to JNK and ERK1/2 upon stimulation with MIP3α. Furthermore, these reports identify that the LFA-1 receptor, when presented with multiple ligands, can result in distinct T cell differentiation states and suggest that the combinatorial integration of ICAM ligand interactions with LFA-1 have functional consequences for T cell biology.

**Conclusion:**

Thus, the ICAM ligands, differentially modulate LFA-1 signaling in T cells and potentiate the development of memory human T cells *in vitro*. These findings are of importance in a mechanistic understanding of memory cell differentiation and *ex vivo *generation of memory cell subsets for therapeutic applications.

## Background

Leukocyte Function Antigen-1 (LFA-1), an αβ heterodimer integrin, is necessary for leukocyte adhesion and migration and is important in the formation of the immunological synapses [1-3]. LFA-1 also has prominent roles in T cell costimulation [4,5] and transendothelial migration [6]. The LFA-1 ligands, intracellular adhesion molecules (ICAMs) -1, -2, and -3, differentially bind to LFA-1 and regulate its adhesion [7-9]. LFA-1 is a mediator of T cell driven inflammatory diseases such as psoriasis[10], rheumatoid arthritis[11], and multiple sclerosis [12], and is a pharmaceutical target for the prevention of the rejection of organ transplantation [13].

Peripheral blood lymphocytes (PBLs) primarily express LFA-1 as opposed to the other beta-2 integrins, such as MAC-1 (CD11b/CD18) and p150,95 (CD11c/CD18), and serves as a primary adhesive molecule for T cells. However, natural killer (NK) cells, eosinophils, neutrophils, monocytes, and dendritic cells also express LFA-1 and other integrins. Impairment of LFA-1/ICAM interactions, using monoclonal antibodies or in LFA-1 knockout mice, disrupts adhesion and migration of neutrophils, monocytes, eosinophils, and NK cells in several disease models of inflammation. Pathologies in these models include thioglycollate-induced peritonitis, delayed delayed-type hypersensitivity, asthma, and susceptibility to bacterial and viral infection [14-16].

Significant advances have been made in understanding the structural properties of LFA-1 and the conformational states that LFA-1 adopts upon ligand binding [17-19]. However, it is not clear how LFA-1 governs interactions in the context of multiple ligands or if LFA-1 signaling mechanisms are similar in different cell types. Previously, we showed that LFA-1 transmitted distinct intracellular signaling events that, in the presence of CD3 and CD28 stimulation, enhanced T cell activation thresholds and polarized T cells towards IFNγ-producing effector cells[20]. Since human effector T cells differentiate into memory T cells, and LFA ligands are known to be important for enhancing the interaction at the immunological synapse, we investigated how the three endogenous LFA-1 ligands influenced commitment of human T cells to adopt a T_H_1 memory phenotype. In particular, it is of interest as to whether the combinatorial integration of signaling induced by LFA-1's interactions with its ligands achieved an internal signaling threshold that committed T cells to end-fate decisions such as differentiation into a particular memory cell subset.

We find that the ICAM ligands differentially promote cell survival. In the presence of CD3 and CD28, stimulation with either ICAM-2 or ICAM-3 suppressed activation of caspase-3. Phospho-proteomic profiling showed that p38 and p44/42 phosphorylation was enhanced and rate of activation was increased. Production of TGFβ1 was decreased when cells were treated with CD3/CD28 plus ICAM-2 or ICAM-3, but not ICAM-1, indicating that different ICAMs can alter cytokine production that can influence T_H_1/T_H_2 T cell development. In long-term differentiation experiments, CD3/CD28 plus ICAM-3 or ICAM-2 generated a higher frequency of IFNγ producing CD4+CD45RO+CD62L-CD11a^Bright ^cells than stimulation with ICAM-1. Only ICAM-3/CD3/CD28 stimulation resulted in differentiation to the CD4+CD45RO+CD62L-CD11a^Bright^CD27- phenotype that transmigrated in response to chemokines. Upon stimulation with MIP3α, the highly differentiated CD4+ memory T cells that were generated by ICAM-3/CD3/CD28 produced a higher frequency of phospho-p44/42 and phospho-JNK positive cells than those stimulated with the other ICAMs, indicating a functionally responsive CD4+ memory cell subset.

These results suggest that signal activation thresholds for CD4+ memory T cell differentiation are integrated with, and bounded by, the combinatorial integration of input signals of CD3, CD28 and LFA-1. However, only ICAM-2 and ICAM-3 showed important responses to the production of highly differentiated functional memory CD4+ T cells in vitro. T cell commitment drives many adaptive immune system processes and thus these results are relevant to situations where multiple ligands interact with a single receptor to drive different underlying processes. The analysis shown here demonstrates that it is possible to distinguish these events at the single cell level.

## Results

### Stimulation of LFA-1 with ICAM-2 or ICAM-3 in the presence of CD3/CD28 co-stimulation does not activate caspase-3

**Figure 1:**
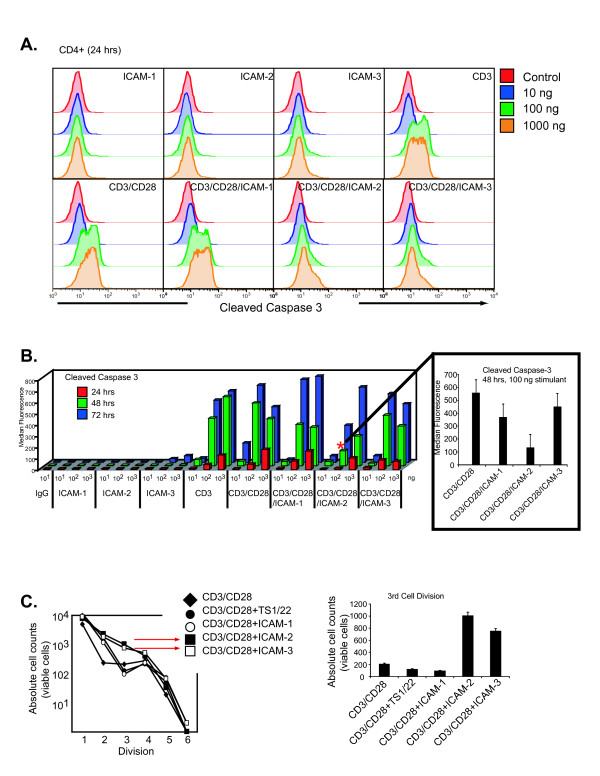
**Stimulation of LFA-1 through ICAM-2 and ICAM-3 in the presence of CD3/CD28 co-stimulation does not activate caspase-3**. (A) Intracellular staining of cleaved-caspase 3 in cells treated with ICAM-1, ICAM-2, ICAM-3, CD3, CD3/CD28, CD3/CD28/ICAM-1, or CD3/CD28/ICAM-2, or CD3/CD28/ICAM-3. Naïve T cells were stimulated in 96-well coated plates and stained for CD4-PercpCy5.5 and intracellular cleaved-caspase-3-PE at 24 hrs. Data is representative of triplicate measures from one donor. Similar results were observed across three donors. (B) Cytometric bead array assessment of cleaved caspase-3 at 24, 48, and 72 hrs after naïve T cells were stimulated with 10, 100, 1000 ng of ICAM-1, ICAM-2, ICAM-3, CD3, CD3/CD28, ICAM-1/CD3/CD28, ICAM-2/CD3/CD28, or ICAM-3/CD3/CD28. Results are averaged from triplicate assays from three donors. Insert displays standard deviation for 48 hour measurement as denoted by the asterisk. (C) Absolute counts of cell divisions of naïve T cells stimulated with CD3/CD28 (control) or in the presence of TS1/22, ICAM-1, ICAM-2, or ICAM-3. T cells were labeled with CMFDA, stimulated in coated 96-well plates, and analyzed at day 7. TruCount beads were used to determine absolute viable cell numbers of divisional peaks detected by CMFDA fluorescence. Assays were performed on 6 individual donors. Triplicate measures from one donor are displayed in insert.

Clonal expansion following T-cell antigen receptor (TCR) stimulation results in T cell activation, cellular division, and under certain circumstances the initiation of activation-induced cell death (AICD)[21,22]. We investigated the effects of the three major LFA-1 ligands on their capacity to enhance cell survival. For these assays, ligand/antibody absorption was optimized on positively charged polystyrene surface 96-well plates to orient globular Ig structures in the proper orientation (see Methods). Additionally, all ligands for stimulation were used as FC constructs for proper orientation. Naïve CD4+ T cells were stimulated with increasing doses of CD3/CD28 in the presence of the different ICAMs. Naive T cells were stimulated with ICAM-1, ICAM-2, ICAM-3, CD3, CD3/CD28 (1:1 protein ratio), ICAM-1/CD3/CD28, ICAM-2/CD3/CD28, or ICAM-3/CD3/CD28 (1:1:1 protein ratio) coated plates at 10, 100 and 1000 ng, and were monitored for the intracellular activation of caspase-3 by flow cytometry at 24 hrs. Stimulation with ICAM-1, ICAM-2 or ICAM-3 alone did not induce caspase-3 activation (Fig. 1A). CD3 and CD3/CD28 alone induced a concentration dependent increase in activated caspase-3, which was similar to CD3/CD28 plus ICAM-1 (Fig. 1A). CD3/CD28 in the presence of ICAM-2 or ICAM-3 stimulation did not activate caspase-3 (Fig. 1A) at any of the tested concentrations at 24 hours. We confirmed these results using a cytometric bead array to measure active caspase-3 at 24, 48, and 72 hrs (Fig. 1B). The expected activation of caspase-3 after CD3 or CD3/CD28 stimulation was observed (Fig. 1B). In the presence of CD3 and CD28, ICAM-2 stimulation resulted in lower levels of active caspase-3 at the 48 and 72 hr time points than ICAM-1 or ICAM-3 stimulations (Fig. 1B).

We investigated whether the reduced cell death induced by ICAM-2/CD3/CD28 correlated with enhanced cellular division. Purified, naïve CD4+ T cells were labeled with 5-chloromethylfluorescein diacetate (CMFDA) and were incubated on CD3/CD28 coated plates in the presence of equivalent amounts of CD3/CD28, ICAM-1/CD3/CD28, ICAM-2/CD3/CD28, ICAM-3/CD3/CD28, or CD3/CD28 plus LFA-1 antibody TS1/22 and were monitored for seven days. We quantified the absolute cell counts in each of the cell cycle divisions using TruCount beads in order to quanitate the viable cells and compare the effect of ICAM stimulation to that of LFA-1 antibody stimulation. A greater numbers of cells in samples treated with CD3/CD28 plus ICAM-2 and CD3/CD28 plus ICAM-3 underwent three cell divisions than cells in samples treated with CD3/CD28 in the presence of ICAM-1, TS1/22, or control (CD3/CD28 alone) (Fig. 1C). The lower numbers of cells entering the third cell cycle division for CD3/CD28 plus ICAM-1, TS1/22, and control samples correlated with higher cell death numbers in those treatments (Fig. 1C). These observations support the notion that the different ICAM ligands induce different cell division and death responses upon LFA-1 engagement in vitro. Consequently, the results suggest that LFA-1 signaling can integrate with that of CD3/CD28 to enhance proliferation and promote cell survival when stimulated with the appropriate ligand.

### LFA-1 transmits intracellular signals that integrate with those of CD3 and CD28

**Figure 2:**
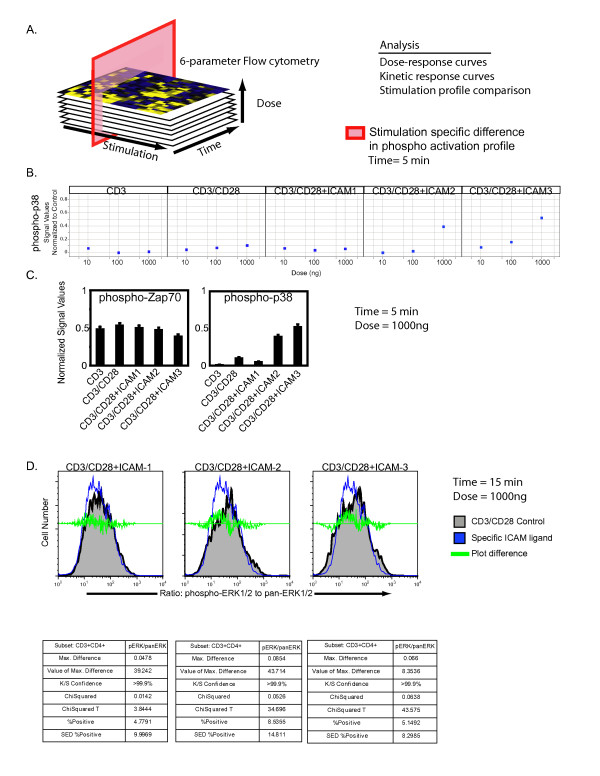
**Phospho-epitope profiling of signaling cascades upon LFA-1 signaling**. (A) Graphical representation of screening strategy. (B) 250,000 naïve CD4+ T cells were crosslinked with CD3 (mAb UCHT1), CD28 (clone 28.2) or ICAM ligand on U-bottom plates for 5, 10, 15, 30 minutes. Adherent cells were fixed, permeabilized, and stained for phospho-epitopes. Data values are relative to non-adherent control cells and were computed using Spotfire software. Data is displayed as a scattered plot profile as a function of the concentration of stimulation agent. Data is averaged from triplicate measures from four individual donors. (C) Phospho-proteomic analysis of p38 and zap70 after 5 minutes stimulation at 1000 ng ligand. Data was computed as the ratio of stimulated to unstimulated cells. Error bars are computed from triplicates. (D) Ratiometric measure of intracellular phosphorylated-p44/42 and pan-p44/42 as a function of CD3/CD28, ICAM-1/CD3/CD28, ICAM-2/CD3/CD28, or ICAM-3/CD3/CD28 stimulation (1000 ng) after 15 minutes. Cells were stimulated in 96-well coated plates and then stained with CD4-PercpCy5.5, phospho-p44/42-PE, and pan-p44/42-AX680. Quantitation of the ratio of phospho-p44/42 to pan-p44/42 was computed in Flowjo. The data is representative from triplicate experiments. The experiment was performed using cells from two donors.

Given that LFA-1 adhesion is an important determinant of outcome at the immunological synapse, we surveyed signaling molecules that serve as points of signal integration with TCR signaling. To identify a nodal point of signal crosstalk between LFA-1 and CD3/CD28, we evaluated the phospho-signature profiles of all three ICAM ligands in the presence of CD3 and CD28. We quantified the phosphorylation status of T cell signaling molecules PLCγ1, Lck, Zap70/Syk, p38, and p44/42 as a function of ligand concentration and time of stimulation by multiparameter flow cytometry (Fig. 2A). We performed both dose and kinetic response analysis to determine stimulation-specific differences. Although rates of phosphorylation were dependent on concentration of ligand as expected, we did not observe significant differences for proximal signaling molecules such as Lck, Zap70, or PLCγ1 (data not shown). At 5 minutes, phosphorylation of p38 was observed to be increased in a dose-dependent manner in ICAM-2/CD3/CD28 and ICAM-3/CD3/CD28 treated samples but not other treatments (Fig. 2B), but no stimulation-specific effect was observed for phosphorylation of Zap70 (Fig. 2C), suggesting that initial kinetics of signal transduction to MAPK proteins was affected differentially by the ICAM ligands. At longer time points, phosphorylation levels of p38 saturated to similar levels in all CD3/CD28 treatment groups (data not shown).

We evaluated the phosphorylation levels of ERK1/2, a kinase essential for T cell activation. Using a ratiometric assay, we measured the levels of intracellular phosphorylated and non-phosphorylated Erk1/2 in populations of T cells stimulated with CD3/CD28 and the ICAM ligands. This assay provides a single cell based quantitative measurement to assess signaling thresholds of ERK1/2. We computationally derived the ratio of the two antibody stains in CD4+ T cells and used statistical algorithms to compare the treatment populations (Fig. 2D). We utilized four different comparison algorithms to determine whether the addition of the ICAM ligands resulted in a statistically significant difference compared to the CD3/CD28 stimulation (Fig. 2D). Two algorithms, the Overton cumulative histogram subtraction algorithm and the Super-enhanced Dmax subtraction (SED), calculate the percentage of positive cells found in the treatment groups relative to the control[23]. More positive cells were found in samples treated with ICAM ligands than CD3/CD28 alone (Fig. 2D). The probability binning (Chi(T)) algorithm was used to compare the ratio of phospho-ERK1/2 to non-phospho-ERK1/2 in samples treated with ICAM ligands to the control[24,25]. This algorithm can detect small differences between two populations. A value of T(X) > 4 implies that the distributions are different with a p value < 0.01[24,26]. The Chi(T) metric T(X) was 3.8 for the ICAM-1/CD3/CD28 treatment, 34.7 for the ICAM-2/CD3/CD28 treatment, and 43.6 for the ICAM-3/CD3/CD28 treatment (Fig. 2D). Therefore, the ICAM-2 and ICAM-3 treatments had ratios of phospho-ERK1/2 to non-phospho-ERK1/2 that were statistically different than that of CD3/CD28 alone. In the presence of CD3/C28, all three ligands induced phospho-ERK1/2 signaling responses over the course of 30 minutes. However, there were differences in both the kinetics and signal intensity of the phosphorylation, supporting our prior findings that LFA-1 can signal to ERK1/2 [20]. These results suggest that p38 and ERK1/2 are two signaling nodes where signal integration from different cell surface receptors occurs. The differences in observed signaling thresholds with ICAM-1 could be correlated with a lack of T cell differentiation (see below).

### Differential capacity of the ICAM ligands to promote CD45RO+ memory cell differentiation

**Figure 3:**
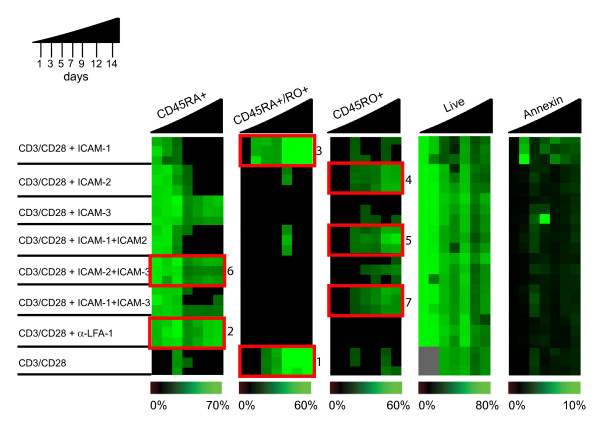
**ICAM-1, ICAM-2, and ICAM-3 differentially promote memory cell development**. Progression of naïve to memory cell differentiation was monitored by immunophenotyping of CD45RA, CD45RO in CD4+T cells over 14 days, with full CD45RO development observed by ICAM-2 stimulation in the presence of CD3/CD28. 5 × 10^6 ^naïve CD4+ T cells were plated on 96-well plates coated with CD3/CD28 (control) or CD3/CD28 in the presence of α-LFA-1 antibody (clone TS1/22), ICAM-1, ICAM-2, ICAM-3, or combinations of ICAMs (as indicated). Cells were stained with CD45RA-PE, CD45RO-Cychrome, Annexin-FITC, and PI on days 1, 3, 5, 7, 9, 12, and 14. TruCount beads were used to obtain absolute cell counts. Data was computed and compiled using TreeView software for pattern recognition. The color intensities are averaged from triplicate experiments and each row represents an individual donor. Triplicate data for each of the three donors is displayed per stimulant. The experiment was repeated with naïve T cells from six donors.

Since LFA-1 signaling can promote early effector cell generation of T_H_1 cells[20], we tested whether the observed differences in signaling thresholds of the ICAM ligands had functional consequences in the long-term differentiation of naïve CD45RA+ T cells to memory CD45RO T cells. We plated naïve CD4+ CD45RA+ T cells on CD3/CD28 coated plates in the presence of equivalent amounts of ICAM-1, ICAM-2, ICAM-3, or α-LFA-1, or combinations of ICAM1/ICAM-2, ICAM-2/ICAM-3, or ICAM-1/ICAM-3. Over the course of 14 days, we monitored CD45RA and CD45RO expression and live/dead cell discrimination by PI and Annexin-V stain. The data was visualized by generation of heatmaps to facilitate pattern recognition. Following stimulation with CD3/CD28 alone a small percentage of cells differentiated to CD45RO+ T cells, but the majority of the cells remained in a CD45RA+CD45RO+ state (Fig. 3, panel 1). Exposure to the α-LFA-1 monoclonal antibody (TS1/22) blocked CD45RA+ progression into the subsequent stages (Fig. 3, panel 2).

However, important differences were observed after stimulation with ICAM-1, ICAM-2, and/or ICAM-3 in the presence of CD3/CD28 co-stimulation. Cells stimulated with ICAM-1/CD3/CD28 progressed to the CD45RA+CD45RO+ stage, but there was a slight increase in detected Annexin-V staining and a decrease in percentage of remaining live cells as compared to cells stimulated with CD3/CD28 only (Fig. 3, panel 3). This however is less than 10% and considered to be in the range of background for apoptotic assays. Few cells progressed to the CD45RO+ stage upon ICAM-1 or ICAM-3 stimulation. Cells stimulated with ICAM-2/CD3/CD28 differentiated to the CD45RO+ stage (Fig. 3, panel 4). Therefore, different LFA-1 ligands resulted in important phenotypic outcomes during *in vitro *progression from CD45RA to CD45RO.

When tested in combination, the ICAMs also showed interesting dominant, synergistic or non-intuitive outcomes. For instance, the combination of ICAM-2 with ICAM-1 in the presence of CD3/D28 enabled the progression to CD45RO+ differentiation, with an apparent dominance of ICAM-2 over ICAM-1 (Fig. 3, panel 5). In contrast, cells treated with ICAM-1/CD3/CD28 did not progress past the CD45RA+CD45RO+ stage (Fig. 3, panel 3). Paradoxically, even though individually ICAM-2 and ICAM-3 in the presence of CD3/CD28 gave the best signaling in our tests of Erk1/2 activation and promoted cell survival, stimulation with both ICAM-3 and ICAM-2 in the presence of CD3/CD28 reduced the frequency of CD45RO+ cells compared to cells stimulated with CD3/CD28 only and blocked progression at the CD45RA+ stage (Fig. 3, panel 6). The combination of ICAM-1 and ICAM-3 with CD3/CD28 also increased the percentage of CD45RO+ cells relative to CD3/CD28 treatment (Fig. 3, panel 7). Thus engagement of LFA-1 with multiple ligands on the same T cell is not the same and leads to critically different biologic outcomes during T cell differentiation. Given the spatial and temporal distribution of ICAM expression on hematopoetic and non-hematopoetic tissues such as endothelium, the activation of T cells at sites of inflammation where ICAMs are upregulated may be modulated by competitive ICAM ligands.

**Figure 4:**
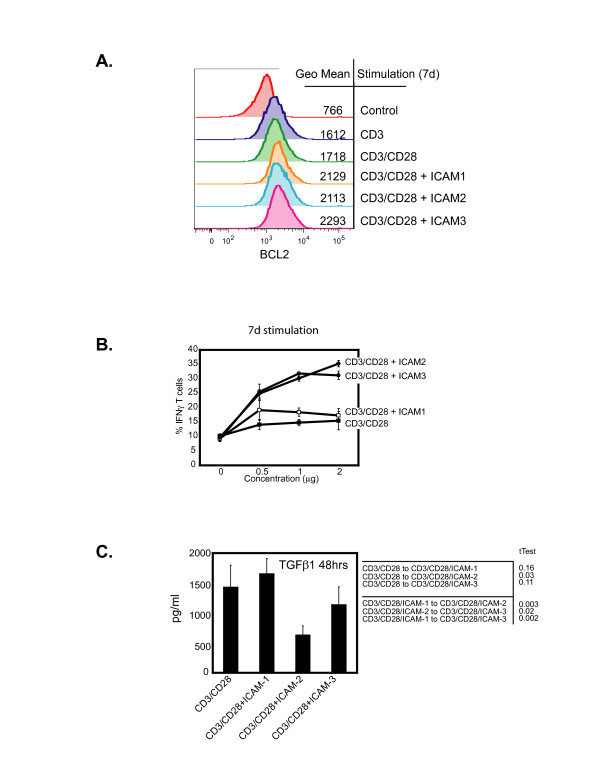
**Distinct functional properties of naïve CD4 T cells stimulated by ICAM ligands in the presence of CD3/CD28**. (A) Intracellular expression of BCL-2 following stimulation of naïve CD4+ T cells with CD3, CD3/CD28, ICAM-1/CD3/CD28, ICAM-2/CD3/CD28, or ICAM-3/CD3/CD28. Mean values for fluorescent intensity are indicated by the histogram. Assay was performed in triplicates from four donors. (B) Frequency of IFNγ producing CD4+T cells after 7 days of stimulation with CD3/CD28, ICAM-1/CD3/CD28, ICAM-2/CD3/CD28, or ICAM-3/CD3/CD28. Assay displays standard deviation from triplicate measurements and was repeated in T cells from 4 different donors. (C) Secreted production of TGFβ1 after 48 hours of stimulation. Graph displays standard deviation from triplicate experiments from two different donors. tTest calculations were performed and indicated in the table insert, with p < 0.05 being significant

Since long-term differentiation requires blockade of cell death programs, we sought to evaluate the intracellular levels of BCL-2, an anti-apoptotic molecule, after a 7 day differentiation period. Expression of intracellular BCL2 was more prominent in CD4 T cells stimulated with the ICAM ligands and CD3/CD28 after 7 days than cells stimulated with CD3/CD28 only; cells stimulated with ICAM-3 exhibited highest levels of BCL2 expression (Fig. 4A). These results corroborate the enhanced early clonal expansion and promotion of cell survival at early stages of activation (Fig. 1) and thus illustrate that the ICAMs can differentially potentiate CD3/CD28 signaling,

We then determined the specific functional differences in the differentiated CD4+ T cells as mediated by the ICAM ligands. Higher frequencies of IFNγ-producing T cells were observed after 7 days in populations treated with ICAM-2/CD3/CD28 or ICAM-3/CD3/CD28 than samples treated with ICAM-1/CD3/CD28 (Fig. 4B). These differences were also observed with extracellular production of IFNγ (data not shown). Therefore, although all stimulations of the ICAMs with CD3/CD28 could promote production of the T_H_1 cytokine IFNγ, there were differences in the ICAMs stimulatory capacity to generate IFNγ producing T cells.

To examine the repetoire of cytokine production as induced distinctly by the ICAM ligands, we profiled the production of 25 cytokines from purified naïve T cells stimulated with CD3/CD28 in the presence of each of the three ICAM ligands over the course of 14 days. It was observed that production of TGFβ1 was diminished when CD3/CD28 was combined with ICAM-2 and to a lesser degree ICAM-3, but not ICAM-1 (Fig. 4C). This was confirmed using T cells from different donors (data not shown). Since TGFβ1 blocks T cell proliferation and differentiation[27,28], the significance of these results could be linked to the generation of functionally distinct T cell memory subsets, a conclusion supported by our observations in Fig. 3, and explored below.

### Generation of CD45RO+ memory T cell subsets with distinct phenotypic and functional characteristics upon stimulation with different ICAM ligands and CD3/CD28

**Figure 5:**
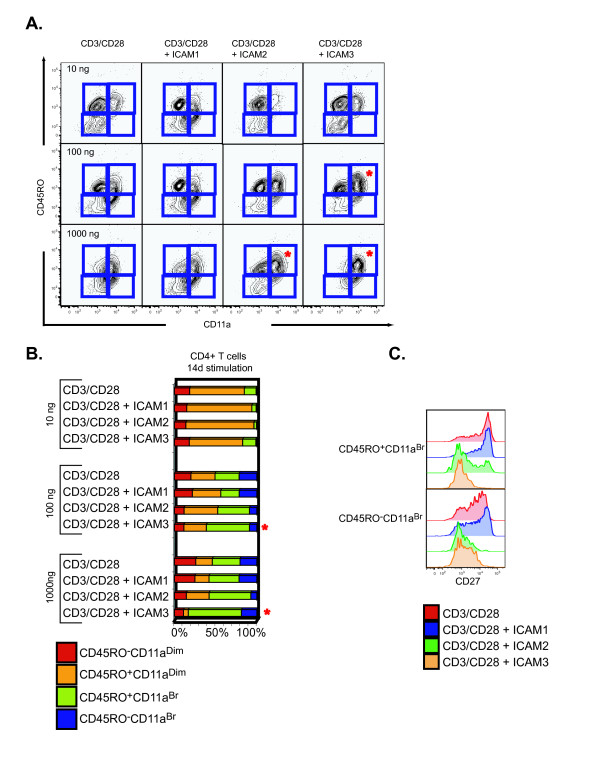
**Distinct phenotypes of CD45RO+ memory T cell subsets generated upon stimulation with different ICAM ligands and CD3/CD28**. (A) Surface phenotype of naïve CD4+ T cells after 14 days of stimulation with CD3/CD28, ICAM-1/CD3/CD28, ICAM-2/CD3/CD28, or ICAM-3/CD3/CD28 at 10, 100, and 1000 ng. CD4+ T cells were assessed for expression of CD45RO and CD11a. (B) Quantitation of the frequency distribution of the generated memory CD4+ T cell subsets was assessed on a fixed acquisition of 10,000 CD3+CD4+ T cells. Gated sections (as presented in Fig. 5A) were computed and are presented in a color-stacked bar graph: CD45RO-CD11a^dim ^(red), CD45RO+CD11a^dim ^(orange), CD45RO+CD11a^Br ^(green), and CD45RO-CD11a^Br ^(blue). (C) CD27 expression of CD45RO+CD11a^Br ^and CD45RO-CD11a^Br ^naïve CD4+ T cells stimulated with CD3/CD28, ICAM-1/CD3/CD28, ICAM-2/CD3/CD28, or ICAM-3/CD3/CD28 for 14 days. Flow cytometry plots are representative of one donor. Assay was repeated with material from 8 donors.

Different ICAM ligands generated phenotypically distinct subsets of CD45RO^+ ^T cells *in vitro *(Fig. 3). We first assessed the expression of CD27 and CD11a to classify memory T cells into CD4^+^CD45RO^+^CD11a^bright^, CD4^+^CD45RO^+^CD11a^dim^, and into CD4^+^CD45RO^+^CD11a^bright^CD27- memory T cells. Four differentiation markers have been shown to accurately reflect memory T cell subsets [29]. After a 14 day differentiation period, we quantified the CD45RO^+ ^cells expressing CD27 or CD11a as these markers stratify the CD45RO^+ ^cells into memory T cell subsets. Fig. 5A illustrates that the CD45RO^+^CD11a^bright ^population is generated to a greater extent in CD3/CD28 plus ICAM-2 or ICAM-3 at both the 100 ng and 1000 ng stimulation dose than CD3/CD28 plus ICAM-1 or CD3/CD28 alone (red starred quadrants). Quantifying for absolute cell counts, we observed a greater frequency of CD45RO+CD11a^bright ^T cells, a highly differentiated subset, in samples treated with CD3/CD28 plus ICAM-3 than other treatment groups, particularly at 1000 ng of ICAM-3 (Fig. 5B). Phenotyping these cells for CD27, an antigen lost during differentiation of naive T cells to highly differentiated memory T cells, showed that only cells stimulated with CD3/CD28 plus ICAM-2 or CD3/CD28 plus ICAM-3 lost CD27 expression (Fig. 5C). These results suggest that the highly differentiated CD4+ T memory cell subset (CD45RO+CD11a^bright^CD27-) was best generated upon long-term stimulation with CD3/CD28 plus ICAM-3.

**Figure 6:**
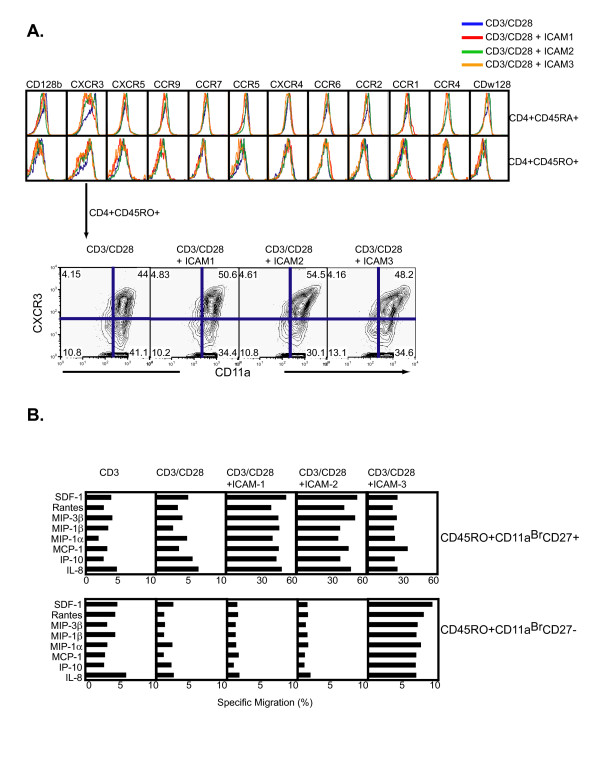
**Generation of CD45RO+ memory T cell subsets with distinct CXCR3 expression and functional characteristics**. (A) Chemokine surface receptor profiling of naïve CD4+ T cells stimulated with CD3/CD28, ICAM-1/CD3/CD28, ICAM-2/CD3/CD28, or ICAM-3/CD3/CD28 for 7 days. Cells were gated on either CD4+CD45RA+ (top row) or CD4+CD45RO+ (bottom row). Highlighted subset contour plots identify expression pattern and frequency for CXCR3 and CD11a on CD4+CD45RO+ cells. Flow cytometry plots are representative of triplicate measures from two individual donors. Repeating assay at lower concentrations of ICAM concentration showed no difference (data not shown). (B) Specific transmigration of naïve CD4+ T cells after 21 days of stimulation. Cells were differentiated in vitro with CD3/CD28, ICAM-1/CD3/CD28, ICAM-2/CD3/CD28, or ICAM-3/CD3/CD28 and subjected to a transmigration assay using specific chemoattractant molecules (as noted in Fig.). After 24 hours, cells were immunophenotyped for CD4+CD45RO+CD11a^Br^CD27+ (top panel) and CD4+CD45RO+CD11a^Br^CD27- (bottom panel). Experiments were reproduced with cells from two different donors. Transmigration experiments were repeated in duplicates with material from three donors. tTest calculations were performed and indicated in the table insert, with p < 0.05 being significant.

This phenotypic analysis was extended to survey a panel of chemokine receptors as chemokine receptors have been recently identified as surrogates of unique terminally differentiated subsets [30]. Of the receptors surveyed, CXCR3 exhibited ICAM specific stimulation expression (Fig. 6A). The frequency of generated CD4+CD45RO+CD11a^br^CXCR3+ cells was greatest with CD3/CD28 plus ICAM-2, however, all ICAM treatments displayed greater frequencies of CXCR3 expression than cells treated with CD3/CD28 alone (Fig. 6A). These results indicate that distinct memory cell subsets, as demarcated by surface markers CD11a, CD27, and CXCR3 can be generated by the ICAM ligands in the presence of CD3/CD28. Importantly, since CXCR3 is a receptor for IP10 (interferonγ-inducible 10 kDa protein), Mig (monokine induced by interferonγ), and I-TAC (interferon-inducible T cell α-chemoattractant), and a major participant in Th-1 induced inflammation [31], the elevated expression in CD3/CD28/ICAM-2 stimulated cells suggest these cells are poised to be responsive to T_H_1 CXC ligands.

**Figure 7:**
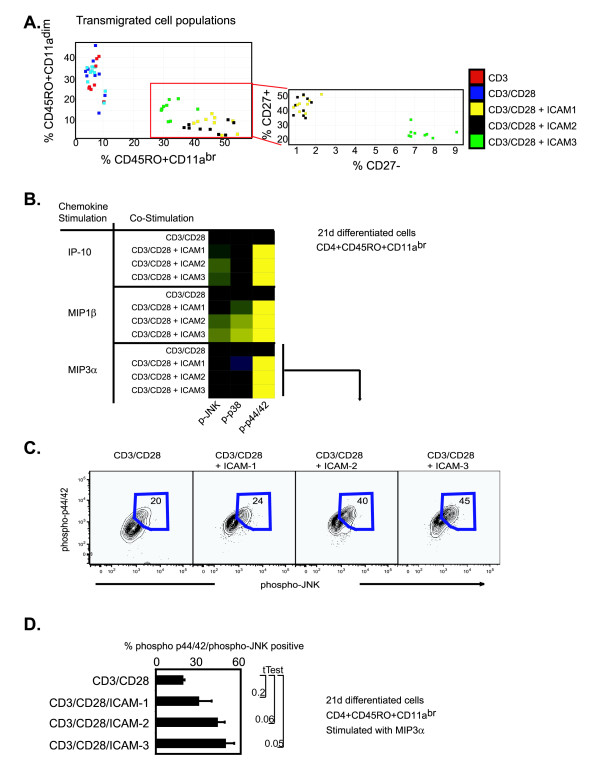
**ICAM ligands induce distinct functional properties in ex vivo generated memory CD4+ T cells**. (A) Analysis of transmigrated memory cell subsets after 21 days of stimulation with CD3, CD3/CD28, ICAM-1/CD3/CD28, ICAM-2/CD3/CD28, or ICAM-3/CD3/CD28. Frequency of subset specific transmigration is presented on the axis. Inset is display of the CD4+CD45RO+CD11a^Dim ^and CD4+CD45RO+CD11a^Br ^populations on CD27 expression. (B) Intracellular activation of phospho-JNK, phospho-p38, and phospho-p44/42 upon IP-10, MIP1β, or MIP3α stimulation (100 ng, 15 min) CD4+ T cells stimulated for 21 days with CD3/CD28, ICAM-1/CD3/CD28, ICAM-2/CD3/CD28, or ICAM-3/CD3/CD28. Fluorescent intensities are shown relative to CD3/CD28 and normalized to a Log2 scale. (C) Phospho-subset frequency for phospho-p44/42 and phospho-JNK populations for the MIP3α stimulated cohort as described above. Experiments were reproduced with cells from two different donors. Transmigration experiments were repeated in duplicates with material from two donors. (D) Quantitation of percent phospho-p44/42/phospho-JNK cells of the 21 day differentiated CD4+CD45RO+CD11a^Br ^subset as stimulated by MIP3α from two different donors.

To gain further insight into the functional capabilities of the long-term differentiated CD4 T cells generated with CD3/CD28 and ICAM ligands, 21 day differentiated cells were subjected to a transmigration assay to determine chemotactic capabilities. The specific migration of each subset was expressed as a percentage of the number of those cells in the original starting population. The migration pattern of CD4+CD45RO+CD11a^br^CD27+ was similar for all chemokines tested (SDF-1, Rantes, MIP3β, MIP1β, MIP1α, MCP-1, IP-10, and IL-8) for the CD3/CD28 plus ICAMs, contrasting the CD3/CD28 treatment alone (Fig. 6B). However, only the CD3/CD28 plus ICAM-3 treatment produced CD4+CD45RO+CD11a^Br^CD27- cells that transmigrated (Fig. 6B). Quantifying the absolute counts of the transmigrated populations identified that only CD3/CD28 plus ICAM-3 produced CD4+CD45RO+CD11a^Br^CD27- that were functionally responsive to chemotaxis for all agents tested (Fig. 7A). Therefore, only the CD3/CD28 plus ICAM-3 stimulation generated a highly differentiated memory CD4+ T cell subset that was functionally responsive to properties exhibited by terminally differentiated CD4+ memory T cells *in vivo*.

To more directly elucidate the underlying molecular mechanisms of the highly differentiated CD4+ memory T cell subsets, we analyzed intracellular phosphorylation of JNK, p38 and Erk1/2 in response to IP-10, MIP1β, and MIP3α. Phospho-p44/42 was induced in all treatments of IP-10, MIP1β, and MIP3α only in the CD3/CD28/ICAMs stimulated cells (Fig. 7B). Phospho-p38 and phospho-JNK was enhanced for CD3/CD28 plus ICAM-3 for MIP1β stimulation in contrast to the other CD3/CD28/ICAMs (Fig. 7B). A higher frequency of dually phosphorylated JNK and p44/42 CD4+CD45RO+CD11a^Br ^cells were observed when CD3/CD28 plus ICAM-3 generated cells were stimulated with MIP3α (Fig. 7C). Intriguingly, cells generated with CD3/CD28 or with CD3/CD28 plus ICAM-1 displayed lower levels of JNK and p44/42 phosphorylated cells in response to MIP3α stimulation than CD3/CD28 plus ICAM-2 or CD3/CD28 plus ICAM-3 stimulations (Fig. 7C). These results were reproduced across different donors (Fig. 7D). These results suggest that ICAM-3 and CD3/CD28 co-stimulation generated a highly differentiated memory T cell subset that is functionally distinct from and differentially responsive to immunomodulatory agents compared to cells generated with either ICAM-1 or ICAM-2 and CD3/CD28.

## Discussions and Conclusion

In this report we present evidence that the ICAM ligands of LFA-1 induce distinct signaling activation events that have functional consequences for human CD4+ memory T cell differentiation in vitro. We undertook a comprehensive approach to dissect the differences amongst cells treated with the ICAM ligands within the context of T cell activation and differentiation. Using multiparameter surface phenotyping in conjunction with intracellular phospho-protein profiling, extracellular cytokine profiling, and chemotactic functional assays, we found that distinct CD4+ memory T cell subsets were generated when cells were stimulated with ICAM-3/CD3/CD28 and ICAM-2/CD3/CD28, and contrasted those generated by ICAM-1/CD3/CD28, or CD3/CD28 stimulation.

Intracellular activation of p38 and Erk1/2 was sensitive to the ICAM ligand used in combination with CD3/CD28 stimulation. Both p38 and Erk1/2 are known to integrate signals to regulate both mitogenesis and differentiation in various cellular systems and these two MAPKS are integral for T cell development, activation, and differentiation [32,33]. Single cell quantitation of the intracellular phosphorylation of p38 and the ratiometric measurement of phosphorylated Erk1/2 revealed that the integration of CD3, CD28 and LFA-1 were dependent on the LFA-1 ligand used for stimulation. We surmise that sub-optimal levels of active Erk1/2 result in the first stages of T cell activation (initial cell division, activation marker expression, and cytokine production), but are not sufficient to dictate full conversion into highly differentiated T cells. This can be concluded since not all ICAM stimulations promoted the full conversion of CD45RA^+ ^to CD45RO^+ ^cells in T cell differentiation assays *in vitro*. Intriguingly, when the ICAM ligands were tested in combination, ICAM-1 and ICAM-2 enabled cell progression to the CD45RA^+^CD45RO^+ ^stage, whereas ICAM-1 stimulation alone did not allow cells to pass the CD45RA^+ ^stage. Also, the combination of ICAM-1 and ICAM-3 enabled some cells in the population to progress to the full CD45RO^+ ^stage, whereas neither ICAM-1 nor ICAM-3 alone allowed progression past the CD45RA^+ ^stage (Fig. 3). These observations suggest a complex regulation of signaling events by LFA-1 dependent on the combination of ligands present. Although ICAM-1 and ICAM-3 have been observed at the immunological synapse in separate studies [34,35], the dynamic interaction of among the three ICAM ligands at the synapse has not been reported. Thus, thee results presented here suggest that the combinatorial integration of ICAM ligand interactions with LFA-1 have important and unexpected functional consequences for T cell biology.

Both ICAM-2/CD3/CD28 and ICAM-3/CD3/CD28 stimulations had higher Erk1/2 signaling thresholds and correlated with less activation of caspase-3 and higher levels of intracellular BCL2 levels than other treatments. These stimulation regimes also generated highly differentiated CD4^+ ^memory T cells that were functionally responsive to chemotactic agents. In murine models, Erk1/2 has been implicated in regulation of both positive and negative selection of developing T cells [33,36-38]. This developmental process is dependent on activation-induced cell death mechanisms that remove autoreactive T cells from the periphery. Improper elimination of autoreactive T cells leads to several forms of autoimmunity[39,40]. Inhibition of programmed cell death is also a prominent feature of various forms of T and B cell lymphomas [41,42]. The relationship between intracellular signaling thresholds and cell death mechanisms in human naïve CD4+ T cells are currently not understood, however, the methodologies employed in this study can be used to resolve the interconnectedness of these two processes.

Activation of CD4 T lymphocytes in the presence of specific cytokines causes differentiation into distinct effector T_H _subsets with different immunoregulatory properties. TGFβ1 is an immunosuppressive cytokine that has been observed to suppress the proliferative response of CD4^+^CD45RO^+ ^lymphocytes [43] and inhibit the production of IFNγ [44]. In our experiments, it was noted that CD3/CD28 and CD3/CD28/ICAM-1 stimulation generated the highest levels of TGFβ1 and consequently, these two stimulation regiments also had the lowest frequency of IFNγ producing T cells (Fig. 4C) and suppressed production of CD4^+^CD45RO^+ ^memory cells (Fig. 5B). Therefore, the enhancement in signaling imparted by LFA-1 upon binding ICAM-2 or ICAM-3 in human CD4+ T cells can attenuate T cell differentiation. Mechanistic understanding on how LFA-1 regulates phosphatase activities may shed some light as to how ligand binding correlates with differential intracellular activities and subsequent cellular outcomes.

A clear understanding of the distinctive tissue distributions of the ICAM ligands, and their roles in determining function of T cells, in the human system has not been completely resolved. ICAM-1 (CD54) has a wide tissue distribution on both hematopoietic and non-hematopoietic cells, can be up regulated upon cellular activation and is viewed as the prominent LFA-1 ligand at the immunological synapse in model systems of cell-to-cell contact and of leukocyte rolling [3]. ICAM-1 is closely related to ICAM-3 (CD50), which is constitutively expressed at high levels on leukocytes and epidermal dendritic Langherans cells and can also be up regulated upon activation on endothelial cells. These two ICAMs contrast with ICAM-2 (CD102), which is broadly expressed on leukocytes and constitutively expressed at high levels on vascular endothelium and is not up regulated upon cellular activation [45]. Interestingly soluble forms of the ICAMs exist in human blood and have been correlated with disease indications [46,47]. The physiological significance of soluble ICAMs in the blood is unknown; however, given the results presented here, one can surmise that the presence of extracellular LFA-1 ligands might potentiate the response of T cell activation and subsequent differentiation.

It is interesting to note that ICAM-1 is the only LFA-1 ligand that has been studied extensively for its adhesive contribution in the immune synapse formation and has been previously implicated to promote increased T_H_1 differentiation [43,48]. From biophysical experiments, ICAM affinity interactions have been calculated using recombinant proteins to suggest ICAM-1 has the highest affinity for the LFA-1 receptor [49]. However, more recent studies have suggested a dynamic conformational change of LFA-1 that has been attributed to the discrepancy of 2D and 3D off-rate measurements and requires adjustment of the ligand and receptor densities to accurately estimate an affinity constant [50].

Because structurally, the ICAMs exhibit geometrical differences, with ICAM-1 being the only ICAM reported to require dimerization for activity [51-54] our data suggest further experimentation is necessary to account for the biological differences observed in human memory T cell differentiation in vitro. At present only one published study using ICAM-1-/- splenic antigen presenting cells has demonstrated a delayed response in generating pathogenic CD4+ effector cells in a murine model of diabetes [55], however a comparative study of the different ICAMs in disease models has not demonstrated. Future work will require follow-up in T cell stimulation by selective ICAM-deficient antigen presenting cells in both murine models and human systems.

When T cells interact with antigen presenting cells (APC), these cells display multiple ICAM ligands at their surface. It has been observed that one T cell can interact with several APCs [56], thereby altering the potential density of locally present interacting LFA-1 ligands. Thus, it is plausible that LFA-1 on any given CD4+ T cell is presented with multiple opportunities to interact with one or more of its ligands, and that the density of ligand interaction governs the intracellular events regulated by LFA-1. This coupled with the dynamic range of *in vivo *peptide-MHC interactions and the number of co-stimulatory molecular interactions warrants further studies into the combinatorial matrix of influential intracellular signaling thresholds that dictate T cell fates. The studies presented here show clearly that interaction simultaneously with different LFA-1 ligands gives rise to different outcomes during memory T cell generation *in vitro*. Thus, cells are capable of interpreting the presence of distinct ICAM molecules presented simultaneously and collating a response that is distinct from their response to any individual ICAM. Whether this is due to action upon all receptors simultaneously or different ICAM ligands, when presented in combination, seek out distinct LFA-1 receptors, with at present unknown modifications or abilities, on the cell surface remains to be determined.

We previously demonstrated that LFA-1 lowers T cell activation thresholds[20] and we recently showed that signaling through LFA-1 can activate human NK cells[57]. The present work demonstrates that LFA-1 signaling mechanisms can potentiate T_H_1 development and that the combinatorial integration of ligand dependent LFA-1 signaling regulates the development of memory T cell development. Our results also illustrate that quantifiable intracellular signaling thresholds, as imposed by LFA-1, can regulate T cell commitments. We expect that such thresholds, like Erk1/2 phosphorylation, serve as signaling checkpoints that regulate T_H_1/T_H_2 development. These may be important in T cell-dependent autoimmune diseases, including rheumatoid arthritis and multiple sclerosis, where aberrant cell activity leads to pathological outcomes. The identity of such checkpoints, and the regulatory kinases and adapter proteins that instruct these processes, may serve as novel areas for pharmaceutical intervention for controlling autoreactive T cells in autoimmune diseases and possibly in T-cell cancers

## Methods

### Immunological and Chemical Reagents

Anti-human CD3, CD4, CD45RA, CD45RO, CD11a, and CD27 direct conjugates (FITC/PE/PercP/PerCPCy5.5/APC/Pe-Cy7/APC-Cy7), cleaved caspase-3-PE, BCL2-FITC, and CD128b, CXCR3, CXCR5, CCR9, CCR5, CXCR4, CCR7, CCR6, CCR1, CCR2, CCR4, CDw128 (all on AX647) were obtained from PharMingen. ICAM-2 mAb and ICAM-2-FITC were from IC2/2 Research Diagnostics. Phospho-specific antibodies to p44/42 (T201/Y202), Gsk3β (Y279), Ikkα (S32/36), PLCγ1 (Y783), Lck (Y505), Zap70 (Y319), p38 (T180/Y182), Stat1 (Y701), Stat3 (Y705), Stat3 (S727), Stat5 (Y694), Stat6 (Y694), and PKA (S114) conjugated directly to Alexa dyes were from BD-Biosciences. TruCount beads were from BD-Immunocytometry systems. LFA-1 antibody clones TS1/22 and TS1/18 were obtained from the Developmental Hybridoma Studies Bank. Protein and chemical reagents used (and vendors) included fluorescein isothiocyanate (FITC, Pierce), Alexa Fluor dye series 488, 546, 568, 647, 680, 700 and CFMDA (Molecular Probes). PMA, ionomycin, and propidium iodide were purchased from Sigma. Recombinant human ICAM-1-FC, ICAM2-FC, ICAM3-FC were from R&D Systems. Recombinant cytokines IL-2, IL-4, IL-6, IL-10, IL-12, IFNγ, and TNFα were obtained from PharMingen. Recombinant chemokines IP-10, MIB1β, and MIP3α were from R&D Systems. Secondary antibodies to mouse and rabbit IgG were obtained from Santa Cruz Biotechnologies. Control treatments consisted of mouse IgG (for antibodies), 1% BSA (for proteins), or 0.01% DMSO vehicle (for chemicals). Secondary crosslinkers were evaluated and optimized for stimulations.

### Cell Culture

Human peripheral blood lymphocytes were obtained by Ficoll-plaque density centrifugation (Amersham Pharmacia) of whole blood from healthy donors (Stanford Blood Bank) and depleted for adherent cells. Magnetically activated cell sorting (Dynal) was used to negatively isolate naïve CD4+ cells for studies as indicated. Human cells were maintained in RPMI, 5% human sera AB (Irvine Scientific), and 1% penicillin-streptomycin glutamate (PSQ). Blood from 42 donors were used for these studies. U-bottom Nunc-Immuno plates with MaxiSorp surface were used for immunology assays. Standard ELISA techniques were used to coat plates, briefly, 200 μL of ligands at concentrations of 0.01–1 μg in PBS, pH7.4 were added to wells and incubated at 4°C overnight. For comparative purposes, stoichiometric ratios of either two or three antibody or ligand were maintained constant by equal mixing prior to adsorption. Excess antigen coating solutions were removed and plates were blocked with complete media for 30 min before being incubated with cells. The MaxiSorp surface is a modified, highly charged polystyrene surface with high affinity to molecules with polar or hydrophilic groups and has a high binding capacity for proteins, including globular antibodies in proper orientation. Maximum binding capacity in a monoloayer is 650 ng/cm^2^.

### Flow Cytometry

Intracellular and extracellular staining was performed as described[58]. Intracellular probes for active kinases were made by conjugating phospho-specific antibodies to the Alexa Fluor dye series as described [58, 59]. Kinetic analyses were performed by direct application of fixation buffer in time-synchronized 96-well plates maintained at 37°C. 200 μL of 2% paraformaldehyde was added to 100 μL of 0.5 × 10^6 ^cells, stimulated as indicated, and the mixture was pipetted up and down three times to ensure even mixing. Plates were kept in a 37°C water bath during the process. Fixation was performed for 10 min at 37°C, and plates were then centrifuged (1500 RPM, 5 min, 4°C) and processed for flow cytometric staining. Flow cytometry data are representative of at least three independent experiments. Figure legends indicate specific replicate and donor repeatability. Intracellular cytokine staining was performed as suggested by manufacturer of intracellular IFNγ-APC stain (BD-Pharmingen). The proliferation assay was performed on sorted naïve CD4+ T cells labeled with 1 μM 5-carboxymethylfluorescein diacetate (CMFDA, Molecular Probes) in PBS for 5 min at 37°C. The mixture was added to coated plates (2 × 10^5 ^cells/well) in RPMI medium 1640 containing 5% human AB sera. After 96 hrs, the cells were stained for CD4 and analyzed. Absolute cell counts were done using TruCount beads. Data was collected on a FACSCalibur (four-color) using Cellquest software or an LSRII (12-color) machine with DiVA software (Becton-Dickinson) and analyzed using Flowjo software (Treestar). Clustering analysis, heatmap visualization, principle component analysis, and scatter plot analysis was performed in Spotfire software. Comparison algorithms used for data analysis were supported by Flowjo. Two algorithms (Overton and SED) were used to calculate the percentage of positive cells found in the sample and not in the control. Two algorithms (Kolmogorov-Smirnov (K-S) and Probability Binning (Chi(T) or PB) were used to determine the statistical difference between samples. The PB algorithm has been shown to detect small quantitative differences between two populations.

### Multiplex bead assays

Cytokine detection was performed using either Cytometric Bead Arrays (CBA, PharMingen) to detect T_H_1/T_H_2 cytokines (IL-2, IL-4, IL-5, IL-6, IL-10 TNFα, and IFNγ) on a FACSCalibur machine or using Beadlyte multiplex kits (Upstate Biotechnologies) to detect (IL-2, IL-3, IL-4, IL-5, IL-6, IL-7, IL-9, IL-10, IL-12, IL-13, IL-15, TNFα, IFNγ, MIP1α, Eotaxin, MCP, and GMCSF) on a Luminex machine. The apoptosis CBA kit was from PharMingen. Protocols suggested by the manufacturers were used.

### Transmigration assay

Cells were placed (0.25 × 10^6 ^cells in 100 μL) in the upper well of 24-well transmigration chambers (5 μm pore, Transwell, Costar Corp.) 100 ng SDF-1, Rantes, MIP3β, MIP1α, MIP1β, MCP-1, IP-10, or IL-8 (in 0.5 μL media) was then added to the lower well. Plates were incubated for 24 hrs at 37°C and cells that migrated to the lower chamber were counted using TruCount beads.

## Authors' contributions

OP conceived of the study and participated in the design and coordination of all the experiments, analyzed the data and drafted the manuscript. DM carried out the flow cytometry assays and participated in the data analysis. GPN helped revise the manuscript. All authors read and approved the manuscript.
